# Highly conductive, printable pastes from capillary suspensions

**DOI:** 10.1038/srep31367

**Published:** 2016-08-10

**Authors:** Monica Schneider, Erin Koos, Norbert Willenbacher

**Affiliations:** 1 Gotthard-Franz-Str. 3, 76131 Karlsruhe, Germany

## Abstract

We have used the capillary suspension phenomenon to design conductive pastes for printed electronic applications, such as front side metallization of solar cells, without non-volatile, organic additives that often deteriorate electrical properties. Adding a small amount of a second, immiscible fluid to a suspension creates a network of liquid bridges between the particles. This capillary force-controlled microstructure allows for tuning the flow behavior in a wide range. Yield stress and low-shear viscosity can be adjusted such that long-term stability is provided by inhibiting sedimentation, and, even more importantly, narrow line widths and high aspect ratios are accessible. These ternary mixtures, called capillary suspensions, exhibit a strong degree of shear thinning that allows for conventional coating or printing equipment to be used. Finally, the secondary fluid, beneficial for stability and processing of the wet paste, completely evaporates during drying and sintering. Thus, we obtained high purity silver and nickel layers with a conductivity two times greater than could be obtained with state-of-the-art, commercial materials. This revolutionary concept can be easily applied to other systems using inorganic or even organic conductive particles and represents a fundamental paradigm change to the formulation of pastes for printed electronics.

Producing electronic devices via printing is preferable over other techniques because it is inexpensive and time efficient due to the easy transition to large-scale production with ready availability of industrial devices. This demand for printable electronics is rapidly increasing with the potential to grow to a $50 billion global market in the next few years^1^. Solution-based deposition has become increasingly popular due to the decrease in material wastage and ambient pressure processing at moderate temperatures leading to lower investment costs over other methods such as physical vapor deposition^2^. Printable electronic products range from microscale devices to large scale units made of either inorganic (e.g. front side metallization of solar cells) or organic material systems (e.g. OLEDs). A large variety of established printing equipment can be used during manufacturing: screen printing, where the ink is pushed through a partially permeable screen; gravure printing or flexography, where the ink is transferred roll to roll; or via drop-on-demand methods like inkjet printing, where the controlled deposition of micro-sized droplets can produce fine structures^3^. Ink formulations have to meet several requirements depending on the printing technique and product application. For example, highly viscous printing pastes are necessary for the screen printing process (0.5–50 Pa∙s), whereas inkjet printing requires much lower viscosities (0.001–0.04 Pa∙s)^3^. Rheological additives, usually polymers, are used to adapt the flow behavior. Furthermore, inks containing insoluble components need a stabilizing agent to avoid sedimentation or agglomeration, such as e.g. alkanethiols on gold nanoparticles^4^. A major disadvantage of these crucial components is the inability to completely remove them in subsequent processing steps (e.g. annealing or sintering)^3^,^4^,^5^,^6^. Residues of the additives can adversely affect the product properties, e.g. by reducing the conductivity. Here we present a novel formulation strategy based on the capillary suspension phenomenon avoiding such impurities. Capillary forces are well known to control the strength of wet granular matter^7^,^8^ and have also been found to affect the dispensing and wetting of granular suspensions on super absorbing surfaces^9^. In capillary suspensions, sedimentation of particles is inhibited by adding small amounts of a secondary liquid to a suspension, which is immiscible with the bulk phase^10^,^11^,^12^. The secondary liquid forms a sample-spanning network of particles and droplets, which prevents particle agglomeration and also dramatically changes the rheological behavior from fluid-like to gel-like or from a weak gel to a strong gel. The viscosity and yield stress of capillary suspensions can be tuned by varying the amount of secondary liquid. Capillary suspensions can be created regardless of whether the secondary liquid wets the solid phase better (pendular state, *θ *< 90°) or worse than the bulk fluid (capillary state, *θ *> 90°), where the contact angle *θ* is the angle made by the secondary fluid against the particles when surrounded by the bulk fluid^13^,^14^. Furthermore, additives become superfluous in this kind of formulation as the capillary network is able to stabilize the particles from sedimentation and also acts as a thickener controlling the rheological and, hence, printing properties. As a further advantage, the secondary liquid can be easily removed by evaporation leading to high product purity. Capillary suspensions were previously shown to increase the edge sharpness and uniformity of coated layers for lithium-ion battery electrode pastes^15^. In the current paper, we demonstrate how these formulations can be used to print fine lines and, more importantly, that the conductivity of the printed and sintered layers is substantially increased compared to the state of the art.

This new formulation route is demonstrated here for nickel and silver, but can be easily adapted to other inorganic particulate systems. Nickel layers have been used, e.g., in multilayer capacitors in order to reduce production costs by replacing the internal electrodes with cheaper base metal electrodes, or as porous electrodes for molten carbonate fuel cells (MCFCs)^16^,^17^,^18^. Pastes based on silver particles are employed in the manufacture of the front side metallization of silicon solar cells as well as other specialized applications. In photovoltaic industries, silver pastes are most commonly applied by screen printing with the aim to print narrow contact lines (≈30 μm)^19^ with increased aspect ratio (height to width ratio) in order to increase efficiency of the solar cell by reducing grid shading losses^20^. As silver is an expensive raw material, but required to achieve the desired conductivity, strong efforts are made to improve paste formulation as well as printing technology aiming at uniform lines with optimized cross-sectional shape thus minimizing silver consumption.

The conductive pastes must be stable when stored despite the large difference in density between the inorganic particles and bulk solvent. Such storage often occurs at rest or under very low shear rates (e.g., mild agitation). Therefore, the inks must either have a high viscosity in the low-shear regime, which decreases the sedimentation rate, or, ideally, by having a yield stress, which directly prevents particle mobility. The sample-spanning particle network present in capillary suspensions is able to fulfill these requirements as can be easily seen from the change in texture shown in Fig. 1a for a nickel in paraffin oil suspension with increasing amounts of added secondary liquid. The secondary liquid here is a mixture of the reducing agent dimethylformamide (DMF) and water (1:1 by volume). The pure suspension, with a solids volume fraction of 20 vol%, is a weak gel. With increasing amounts of the secondary liquid mixture (3–10 vol%), the weak gel changes to a strong and stiff gel with a clearly different texture. This textural difference is indicative of a high yield stress where the sample-spanning network is able to hold the weight of the sample and prevent particle sedimentation during, e.g., storage. Long term stability of capillary suspensions was investigated in previously published preliminary experiments, which showed that the otherwise very strong stability is affected when the samples are exposed to high temperatures (~100 °C) for several days^12^. In this case, the capillary network collapses due to the evaporation of the secondary fluid. Nevertheless, the original sample properties, e.g. yield stress, were easily regained after re-adding equivalent amounts of secondary liquid. This is a major advantage when compared to conventional conductive pastes, where aging issues are linked to the degradation of polymeric additives after which the sample properties cannot be easily recovered. Typical storage temperatures of ~10–30 °C do not affect the long term stability as the secondary liquid remains encapsulated by the organic bulk fluid.

Nickel and silver particles were dispersed in terpineol, a solvent commonly used in printing pastes for front side metallization of solar cells, to systematically study the change in flow behavior due to an added secondary liquid. The employed solids volume fraction of 29 vol% (equivalent to about 82 wt% Ag or 80 wt% Ni), is in the range of the solids fractions commonly applied in such pastes (60–90 wt%)^21^. Adding the secondary liquid, distilled water, leads to a strong increase in the suspension yield stress *σ*_*y*_ as can be seen in Fig. 1b. The yield stress of the pure suspension is 5 Pa for silver and 1 Pa in case of the nickel. This value increases by 400-fold for nickel and 140-fold for silver when adding 7 vol% and 5 vol%, respectively, of secondary liquid as measured to the total liquid volume. Distilled water does not preferentially wet the nickel or silver particles in terpineol, forming contact angles of 130° and 156° placing these mixtures in the capillary state. An example of a capillary state network system is shown in the inset to Fig. 1b. Clusters of fluorescently labelled glass beads including small droplets of secondary fluid are visible in this confocal microscopy image obtained using an index-matched paraffin oil as bulk fluid and water (including a yellow fluorescent dye) as secondary fluid^22^. The secondary liquid in these capillary state suspensions forms a sample-spanning network between particle clusters encasing small secondary liquid drops as evident from the strong increase in yield stress with increasing amount of secondary liquid^11^. The capillary network allows for a stabilization of the particles without additional polymeric stabilizers, which are commonly used in other paste formulations^23^,^24^, maintaining homogeneous mixtures during storage. Experiments with other systems have shown that capillary suspensions remain stable for long periods of time, even months, without phase separation^25^.

The strength of a capillary network is directly linked to the interfacial tension between the bulk and secondary liquid^10^. As such, a variation of the secondary liquid, including e.g. solvent mixtures, opens another possible route to adjust the flow behavior in order to meet the requirements of the desired printing technique or to improve the quality of the print in addition to varying the solids loading. In order to demonstrate the universality of the capillary suspension phenomenon, experiments were conducted with different solvent combinations. Figure 2a shows the influence of the secondary liquid for nickel capillary suspensions with a solids fraction of 20 vol% in paraffin oil and three different secondary liquids: pure DMF, a DMF and water mixture (1:1 by volume), and pure water. The corresponding interfacial tension data for these combinations can be taken from Table 1. The yield stress *σ*_*y*_ of a capillary suspension is given by


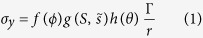


where the strength is linearly proportional to the interfacial tension Γ, the reciprocal particle radius *r*, and depends on various functions for the solids volume fraction *ϕ*, the secondary liquid amount *S*, the normalized particle separation 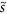, and the three-phase wetting angle *θ*. The function 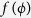 depends on the particle volume fraction *ϕ* and includes the number of capillary bridges per particle. The function 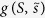 depends on the amount of the secondary liquid *S* and the reduced particle separation 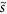 and includes the dependence of the network strength on the size and shape of the liquid bridges. The function of the contact angle, *h*(*θ*), is typically predicted to be cos *θ* in the pendular state, but is more complex in the capillary state^11^. According to the supplier, the particle diameter of the nickel particles used here ranges from 3–7 μm, which is a typical size range for particles used in pastes for printing silver or nickel electrodes^18^,^26^. In this study, DMF has the lowest interfacial tension of 6.0 mN/m, followed by the aqueous DMF mixture (15.6 mN/m). Pure water forms the highest interfacial tension with 48 mN/m in the presence of paraffin oil. This trend in interfacial tension corresponds directly to the measured yield stress, i.e. network strength. Pure H_2_O provides the steepest increase of the normalized yield stress when increasing the secondary fluid amount. For the case of the DMF/H_2_O mixture, a maximum in network strength is reached with 7 vol% added liquid followed by a plateau of the yield stress. The pure DMF also has a maximum in the normalized yield stress at 7 vol%, but shows a loss in network strength at higher added fluid contents. The yield stress values for the aqueous DMF capillary suspensions are higher than the pure DMF capillary suspensions at each secondary fluid content due to the higher interfacial tension. This direct influence of the interfacial tension Γ can also be seen in the inset to Fig. 2a showing the yield stress *σ*_*y*_ normalized by the Laplace pressure Γ/*r*, where *r* is the mean particle radius and constant for the depicted material systems. The proportionality between yield stress and reciprocal particle radius has been confirmed in a previous study^12^.

The data shown in Fig. 2a overlap at low secondary fluid content. In this regime the yield stress is directly proportional to Γ/*r* and can easily be tuned by appropriate choice of secondary liquid and hence interfacial tension. However, the DMF and DMF/H_2_O data diverge at higher amounts of added secondary fluid. This divergence might be due to differing cluster structures presumably occurring at higher secondary fluid content^11^.

During the application or coating step, the inks are subjected to high shear rates that vary strongly with the chosen printing technique (e.g., screen printing 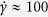 s^−1^, dispensing 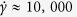
s^−1^) followed by a deformation rate close to zero when the ink settles on the substrate^27^,^28^. Therefore, the inks are required to exhibit low high-shear viscosity that allows the ink to easily flow when passing through the screen mesh or dispensing nozzle during application while also possessing a high low-shear viscosity and a fast network recovery providing good shape accuracy to the printed structure. Figure 2b shows the rheological response of the capillary suspensions containing nickel particles (29 vol%) to different shear rates. Measurements at shear rates below 100 s^−1^ were performed in a rotational rheometer and higher shear rates (up to 10^4 ^s^−1^) were achieved using a capillary rheometer. The viscosity *η* is strongly dependent on the amount of secondary liquid in the low shear regime (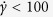
s^−1^). Comparing *η* at 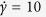
s^−1 ^shows an increase of one decade for 7 vol% of secondary liquid when compared to the pure nickel suspension. All of the samples shown here demonstrate shear-thinning behavior. In the high shear regime (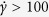 s^−1^), the viscosity functions of the capillary suspensions begin to coincide and approach a plateau value 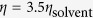 that is predicted for the given particle loading of 29 vol% using the phenomenological model of Quemada^29^. This plateau is expected since the viscosity at these high shear rates should be determined solely by the solids volume fraction and demonstrates that the capillary network is completely broken. The pure nickel suspension is not stable at very higher shear rates, leading to phase separation during the capillary rheometry measurements, and these data are excluded from the figure.

Capillary suspensions exhibit a high yield stress and pronounced shear thinning. The clear advantage of this unique flow behavior in comparison to the pure metal particle suspension is demonstrated in an application oriented printing test, corresponding results are shown in Fig. 3. A top view on the wet contact lines, taken directly following the application of the capillary suspension with terpineol continuous phase on a glass substrate with a doctor blade and 300 μm wide slot stencil, is shown in Fig. 3a for the nickel suspension and 3c for the silver suspension, each with a solids fraction of 29 vol%, with an average maximum line height of 190 ± 27 μm. The top images show the line widths obtained for the suspensions without any secondary liquid and the bottom with added water. Figure 3b,d show the cross sectional height profiles for nickel and silver capillary suspensions, respectively, normalized to their maximum height. Profiles are plotted as function of the x-position from the center, thus the distinct changes in shape are clearly visible. The pure suspensions both spread on the substrate far beyond the 300 μm mask (denoted by the vertical lines in the images) with the nickel suspension even demonstrating spreading of the pure solvent beyond the borders of the paste due to the limited stability, i.e. phase separation. For the nickel capillary suspension with 5 vol% added water, less solvent spreads under the glass stencil. Thus solvent loss is reduced and the line width decreases from 1008 ± 163 μm with no added secondary liquid in the sample to 500 ± 37 μm. The error intervals given here and in the subsequent part of this section reflect the variation of the line width along the printed line, i.e. y-direction. The line width further drops to 470 ± 28 μm and the height profile changes from bell-shaped to almost rectangular with a flat top and minimal spreading when 7 vol% of water is added. Hence, the addition of the secondary liquid leads to a decrease of the line width and an improved uniformity of the profile along the direction of the printed line. The dip of the profile with 5 vol% added liquid close to the sample edges are attributed to measurement errors: Surface topology of the crack-free wet lines has been measured with a 3D laser scanning microscope. Negative values can occur due to the reflection of the beam from the bottom interface or imperfections within the substrate instead from the top interface of the glass substrate. This type of error, due to the sharp discontinuity in reflection, occurs at the edge of the printed line and reflective substrate.

A similar correlation between amount of secondary liquid and line width is found for the silver samples (see Fig. 3c,d). Adding just 3 vol% of water to the silver suspension reduces spreading and the line width decreases from 644 ± 143 μm to 322 ± 30 μm. Moreover, the cross section changes from bell-shaped into a rectangular shape. The required lower amount of secondary liquid corresponds to the higher yield stress of the silver suspensions at a given amount of added liquid compared to the nickel system. Additionally, Fig. 3d shows the profile of a commercial silver sample (Heraeus I) obtained under similar application conditions. The rectangularity (area under the profile compared to the minimum bounding rectangle) of this profile is 0.74 and the corresponding value for the capillary suspension with 3 vol% added water is 0.67. This demonstrates that the capillary suspension formulation is competitive to the conventional formulation with regard to shape accuracy, but the sharp rectangular profile in the capillary suspension is achieved at lower particle loading compared to the commercial paste and without non-volatile organic additives. Such a rectangular line profile increases the cross-sectional area while maintaining a small footprint. This should result in a high conductivity when printing, e.g. small scale circuit boards with fine lines.

Nickel as well as silver capillary suspensions with different amount of secondary fluid were coated on quartz glass substrates and sintered at 800 °C in order to compare their electronic properties with those of model Ni and Ag suspensions including different binders and thixotropic agents as well as commercially available silver pastes. The heating rate in the sinter oven was 15 K/min, the fastest heating rate achievable, in order to simulate the firing profile in a belt furnace during photovoltaic production. Variation of the sintering temperature was not performed here. A change of the sintering profile can affect the resulting electronic properties, e.g. an increase of the maximum sintering temperature results in lower bulk resistivity values for silver based systems^26^, but we assume this effect is similar for the different pastes investigated here. The resulting sheet resistivity *ρ*_*s*_ after annealing was measured using the Van-der-Pauw method and used to calculate the bulk resistivity 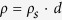 together with the corresponding sheet thicknesses *d* after annealing. Corresponding data for the systems mentioned above are displayed in Fig. 4^30^,^31^. The bulk resistivity of nickel based capillary suspensions along with two other commercial formulations are shown in Fig. 4a and the silver based suspensions and two commercial silver screen printing inks from Heraeus Precious Metals (denoted as Heraeus I and II) are shown in Fig. 4b. The capillary suspensions prepared with nickel and silver show no distinct trend with increasing amount of secondary liquid. The average bulk resistivity of the nickel capillary suspensions is 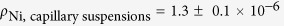
Ωm and 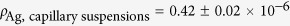 Ωm for the silver capillary suspensions. Obviously, the addition of secondary liquid to the wet suspensions does not affect the electronic properties of the produced nickel and silver layers. We can, therefore, ensure stability and tune the rheology of the paste to meet the demands of different printing methods without significantly varying the conductivity. However, these capillary suspension formulations clearly differ from conventional formulations that include stabilizers, binders and rheology modifiers. The conventional nickel suspensions were prepared following a patent for the formulation of an organic vehicle (abbreviated as ov in the figure) that is used for silver based screen printing pastes, but also suggested for other metal particles such as nickel^21^. The bulk solvent in these formulations was the same as used in the capillary suspensions (terpineol). To the pure nickel-terpineol suspension, polyvinyl pyrrolidon (PVP) is added as an organic binder and surfactant, and ethyl cellulose is added as an additional binder or thickener as described in more detail in the experimental section. Two common thixotropic agents are then added to this organic vehicle: the hydrophobic Aerosil^®^ R805 or the hydrophilic Aerosil^®^ 150^32^. The pastes were prepared following the patent (as is described in the experimental section) and were subjected to the same heat treatment used for the capillary suspensions. The bulk resistivity of these conventional formulations is increased by a factor of two (2.8 ± 1.0 × 10^−6 ^Ωm) for the Aerosil 150 and threefold for the Aerosil R805 formulation (3.5 ± 0.2 × 10^−6 ^Ωm) compared to the nickel capillary suspensions (Fig. 4a) despite having the same loading of nickel particles. The higher bulk resistivity may be attributed to the interference of additive residues in the organic vehicle as has been previously reported^3^,^4^,^5^,^6^. This is supported by sheet thickness measurements shown in Fig. 5. Figure 5a shows the sheet thicknesses of the nickel based suspensions. The capillary suspensions exhibit an average sheet thickness of 185 ± 13 μm and the thickness of the conventional nickel formulations is about 10% higher according to the increased total solids volume caused by the addition of the non-volatile additives that remain in the layer even after sintering. (The volume fraction of nickel is 29 vol% and is the same for both the capillary and conventional formulations).

A similar difference in bulk resistivity is found when silver capillary suspensions are compared to commercial silver pastes including additional components, e.g. thixotropic agent, polymeric stabilizer and other additives (Fig. 4b). The size of the silver particles (≈10 μm) was chosen similar to that of similar as in the commercial silver pastes investigated here. The two commercial formulations, Heraeus I and II exhibit a bulk resistivity 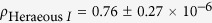 Ωm and 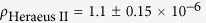
Ωm, respectively, that is about double the value of the silver capillary suspensions when coated and sintered under similar conditions. Presumably, the additives in the commercial pastes do not evaporate or decay completely during the firing step and diminish the conductive properties of the silver layer. The additives may have an additional effect on the structure of the sintered layer here. The capillary silver suspensions exhibit a substantially lower sheet thickness (123 ± 5 μm) than corresponding nickel samples (185 ± 13)μm. With a much higher melting point of 1455 °C (961 °C for silver), the nickel samples do not experience as strong a densification as the silver samples during the applied sintering procedure. However, the commercial silver pastes exhibit a sheet thickness even higher than the nickel samples and this cannot solely be attributed to the remaining additives or the different particle loading. We estimate an upper limit of silver content of 44 vol% based on the solids loading reported by the supplier assuming that the paste consists of silver and terpineol. Accordingly, a sheet thicknesses of 180 μm is expected for the conventional silver formulations, but the measured values of 242 μm (Heraeus I) and 293 μm (Heraeus II), as shown in Fig. 5b, far exceed this predicted value. Therefore, we hypothesize that the additives prevent a complete collapse of the particle network during sintering. Such a particle network would have fewer particle contacts than the denser capillary suspension layer and, accordingly, a lower conductivity, i.e. higher resistivity.

Finally, a silver capillary suspension with 5 vol% added water was prepared containing an additional 6 vol% solder glass (total solids loading 33.4 vol%), which is a crucial component during the firing step in the photovoltaic production for proper contact with the underlying substrate. This additional solid phase does not interfere with the formation of the capillary network and led to an even higher yield stress when compared to the silver capillary suspension with 29 vol% silver and 5 vol% added water. The bulk resistivity increases slightly due to the solder glass (from 0.45 ± 0.05 × 10^−6 ^Ωm to 0.55 ± 0.19 × 10^−6 ^Ωm), but is still much lower than for the commercial Heraeus formulations. We are able to decrease bulk resistivity by more than a factor of two while maintaining an equivalent stability and contact with the substrate as the commercial formulations.

In summary, we have shown that the capillary suspension concept can be used to stabilize inorganic particles used in conductive pastes for printed electronic applications without using polymeric or other non-volatile organic additives, which can remain in the sintered part thereby reducing conductivity. Capillary suspension-based silver and nickel pastes suitable for screen printing resulted in, at least, a twofold increase in conductivity than the corresponding sintered layer compared to conventional, commercial pastes processed under similar conditions. The yield stress of these capillary suspensions can be tuned to provide stability of the pastes during storage, processing and application. The degree of shear thinning can be adjusted to enable easy flow through printing meshes or nozzles. This tunable rheology is demonstrated here using both nickel and silver particles with a combination of bulk solvents and secondary fluids, but can be applied other combinations using inorganic or even organic conductive particles. We have also shown that the unique flow behavior results in a high shape accuracy of the printed pattern. Lines with nearly rectangular cross section are demonstrated. Such lines, with a high aspect ratio, are desired for small scale circuits and even more complex, e.g. triangular shapes advantageous for the front side metallization of solar cells, may also be possible. In addition to these benefits directly related to the unique flow behavior, the capillary suspension concept offers a significant advantage in printed electronic applications due to the lack of non-volatile organic additives. This high purity formulation results in a denser layer with higher conductivity in comparison to conventional formulations.

Beyond the significant improvements demonstrated here in key areas, the capillary suspension concept offers additional opportunities particularly relevant for printed electronic applications. A wide variety of particle sizes and shapes in addition to possible solvent combinations can be used. This offers not only an additional degree of freedom to obtain a desired flow profile, admixtures of micron and sub-micron sized particles may be used to achieve a denser particle packing, i.e. increased grain contact area, leading to an improved conductivity^33^,^34^,^35^. Finally, the secondary fluid may be chosen to be a highly efficient reducing agent (e.g. DMF) that can selectively remove oxidized surface layers in the particle contact regions. We have shown that the capillary suspension concept is an innovative formulation platform ideally suited to design pastes for printed electronic applications with improved processing and conductivity properties. This formulation method requires less expensive ingredients (e.g. water rather than specialized polymers) and offers a promising pathway to increase the conductivity even beyond the twofold increase shown here. The proposed high conductivity formulation concept may even enable silver to be replaced, e.g. in large scale applications like solar cells, by more abundant conductive materials such as copper in the future.

## Materials and Methods

**Figure 2:**
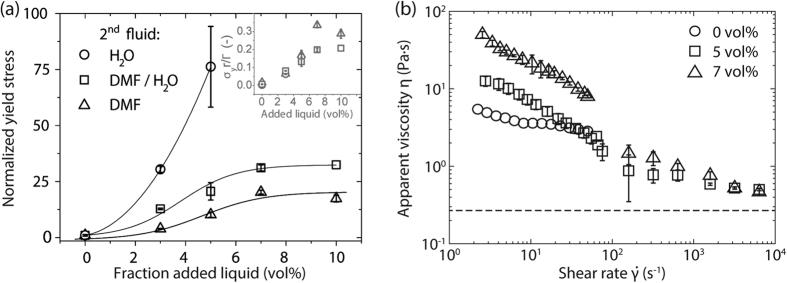
**(a)** Normalized yield stress as function of secondary fluid content for nickel capillary suspensions with paraffin oil as bulk fluid and different secondary liquids: DMF, DMF/H_2_O-mixture (1:1 by vol.), and pure H_2_O. The inset shows the yield stress 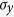 normalized by the Laplace pressure (Γ/*r*). The solids volume fraction for all material combinations shown is 20 vol%. Lines are drawn to guide the eye. **(b)** Viscosity *η* of nickel (29 vol%) in terpineol suspensions as function of shear rate 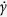 for varying water fractions. The dashed line shows the estimated high shear viscosity for the given solids loading assuming hard sphere particles^29^.

To create the capillary suspensions, the metal particles were first dispersed into the appropriate nonpolar solvent and then water or a mixture of water and DMF was added. Details about the combinations used are summarized in Table 1 and details about the particles and solvents in Table 2. The particle quantity, specified by the desired volume fraction, was added to the bulk phase while slowly stirring with a turbulent beater blade. After adding the solids, the stirrer speed was increased to 1000 rpm for 20 minutes to ensure homogenization. Finally, the capillary network formation was induced by adding the secondary liquid volume with a pipette and stirring at high speed for another 5 minutes. An additional sample using a combination of silver particles and solder glass was prepared as above where the volume of silver particles was supplemented by an additional 6 vol% solder glass. The samples were prepared using distilled water with the exception of the data shown in Fig. 2a, which were also prepared using DMF and a DMF/water mixture.

To compare the rheological and electronic properties of the capillary suspension-based products with conventional formulations, additional silver and nickel samples were used. The silver samples, obtained from Heraeus Precious Metals (Hanau, Germany), are referred to as Heraeus I (SOL9610A, 90.5 ± 1 wt% solids) and Heraeus II (SOL9020C, 90 ± 1.5 wt% solids). Additionally, two nickel samples were prepared following a patent for an organic vehicle^21^. This organic vehicle contained 60 wt% terpineol as solvent. To this solvent, a polymeric stabilizer (5 wt% polyvinylpyrrolidone K30, average M_w _= 40,000 g/mol, Sigma-Aldrich, Steinheim, Germany), a dispersing agent (20 wt% TEGO Dispers 656, Evonik, Essen, Germany), a cellulose based binder (10 wt% sodium carboxymethylcellulose CRT 200PA, DOW Europe GmbH, Bomlitz Germany) as well as 5 wt% dispersing and thixotropic agent with either hydrophilic (Aerosil 150, Evonik, Essen, Germany) or hydrophobic properties (Aerosil R805, Evonik) were added. The components were homogenized by slowly stirring with a turbulent beater blade (370 rpm) at a constant temperature of 70 °C for two hours. After preparation, the organic vehicle was cooled to room temperature. The nickel particles (Alfa Aesar, Nickel powder 3–7 μm, Karlsruhe, Germany) were added to the organic vehicle and the sample was stirred for another 30 minutes at 400 rpm. The nickel particles and volume fraction (29 vol%) were the same as in the capillary suspensions.

The three phase contact angle *θ* was used to characterize the solid-liquid-liquid system and the network type of the capillary suspensions. The contact angle of the nickel capillary suspensions was determined using a sintered nickel film placed in a glass container and covered with the bulk fluid. A droplet of distilled water was placed on the surface of a film using a syringe and the resulting three phase contact angle *θ* of the secondary fluid against the solid was evaluated via image analysis (Krüss, Drop Shape Analysis, Hamburg, Germany). Surface roughness of the nickel film was negligible compared to droplet size. For the contact angle in silver capillary suspensions, the nickel film was replaced by a smooth commercial silver foil (Dukatshop, silver foil 999/1000 purity, Berlin, Germany). The three phase contact angles were all greater than 90° (130° for nickel particles in terpineol with added water and 156° for silver) placing these suspensions in the capillary state.

The yield stress of each suspension was determined using a rotational rheometer (Mars II, Thermo Scientific, Karlsruhe, Germany) with a vane geometry (10 mm diameter) placed in a cylindrical cup (Z20 DIN). The temperature was 20 °C for all measurements. By applying successively increasing stress values and measuring the resulting deformation, two distinct linear regimes are visible in a double-logarithmic plot. The yield stress is defined as the intersection of the two linear curve fits. Flow curves in the low-shear regime were obtained using a sandblasted aluminum plate (35 mm diameter) and strain-controlled measurement procedure. Increasing strain values were applied stepwise and the resulting shear rate measured after a waiting time of 30 seconds. Viscosity measurements at higher shear rates were completed using a modified Göttfert Rheograph 2000 (Göttfert Werkstoff-Prüfmaschinen GmbH, Buchen Germany) capillary rheometer (capillary length: 40 mm, diameter: 1 mm). The pressure difference measured between sample chamber and capillary outlet was measured with a 200 bar pressure transducer (Gefran, Seligenstadt, Germany) at each selected piston speed and is correlated to the shear stress. The high-shear viscosity can then be determined using this shear stress and the shear rate (related to the flow speed). Further measurement corrections, e.g. for wall-slip, were not performed. Therefore, the high-shear data should be treated as apparent viscosity values rather than the true viscosity of the sample.

Films with a defined area were created in order to measure the sheet resistivity resulting from the different formulations via doctor blade. The doctor blade gap height was 300 μm and accurate shapes of 1 cm^2^ were achieved by confining the pattern with a 60 μm thick adhesive tape that was removed promptly after film application. The suspensions were coated on heat stable quartz carriers (PELCO Quartz Substrate Discs, Ted Pella Inc., Redding, Calif.). The nickel and silver films were both sintered at 800 °C with the fastest available heating rate of 15 K/min in order to imitate the temperature profile of a belt furnace, which is usually utilized in solar cell production. After arriving at the maximum temperature, the samples cooled down with purely passive cooling. A nitrogen atmosphere was employed to sinter the nickel samples in order to avoid any further oxidation during the sintering step. Ambient air was used for the silver samples. Sheet resistivity measurements were completed following the Van-der-Pauw method using a self-constructed measurement set-up^30^, starting from an injected current of 0.02 mA and gradually increasing this value up to 0.1 mA. To ensure a good contact between the probes and nickel samples, small droplets of a commercial fast-drying silver paste (Electrodag 1415M, Acheson Colloiden B.V., Scheemda, Netherlands) were placed at each corner of the nickel film. The sheet thicknesses after the sintering process are required for the evaluation of the sheet resistivity (to avoid any errors due to the different thicknesses) and were measured with a 3D laser microscope (VK-X100 Laser Microscope, Keyence, Neu-Isenburg, Germany). For printing of fine lines, the adhesive tape was replaced by a glass stencil (171 μm thickness) with a 300 μm line width. After squeezing the sample through the stencil with a blade, the stencil was carefully removed and the height profile examined with the 3D laser microscope.

## Additional Information

**How to cite this article**: Schneider, M. *et al*. Highly conductive, printable pastes from capillary suspensions. *Sci. Rep*. **6**, 31367; doi: 10.1038/srep31367 (2016).

## Figures and Tables

**Figure 1:**
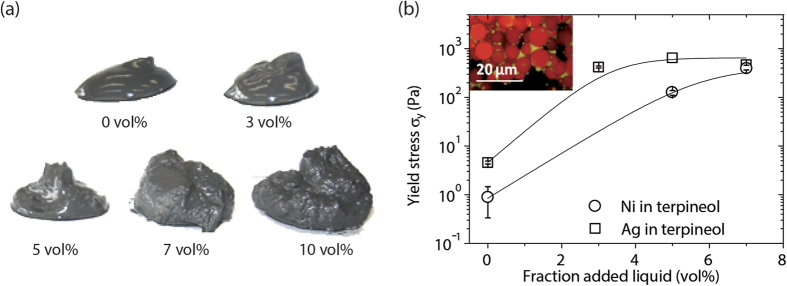
**(a)** Nickel particles in paraffin oil (20 vol% solids loading) with increasing amounts of water-DMF mixture as a secondary liquid. The samples change from a weak gel without added fluid to a strong, stiff gel with added secondary liquid due to the formation of a capillary state network. **(b)** Increase in the yield stress 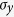 for nickel and silver particles dispersed in terpineol with increasing amounts of distilled water. Both systems are in the capillary state and have a solids volume fraction of 29 vol%. Lines are drawn to guide the eye. The inset shows a confocal scanning laser miscosope image of a ternary particle-liquid-liquid system in the capillary state. Fluorescently labelled glass beads (red) are suspended in paraffin oil and clusters of added secondary fluid are visualized using a yellow fluorescent dye (adapted from^22^).

**Figure 3:**
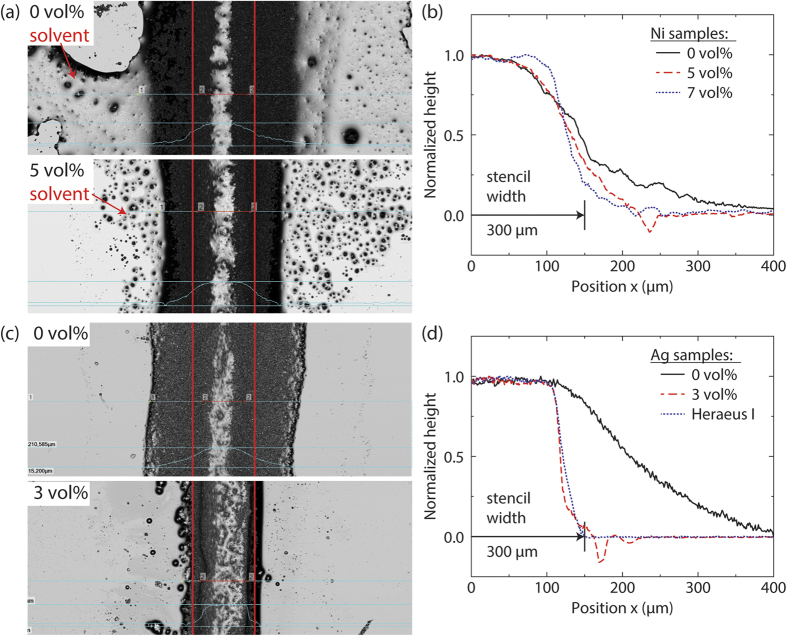
Wet contact lines for **(a)** nickel and **(c)** silver suspensions using a mask of width 300 μm (red vertical lines). The light grey areas and black spots are from the pure solvent, where the contrast is controlled by the drop height. Cross sectional profile for lines printed with **(b)** nickel and **(d)** silver capillary suspensions. All suspensions were prepared in terpineol with varying amounts added water and have a solids fraction of 29 vol%. Additionally, a commercial silver sample (Heraeus I) is plotted for comparison with the silver capillary suspensions.

**Figure 4:**
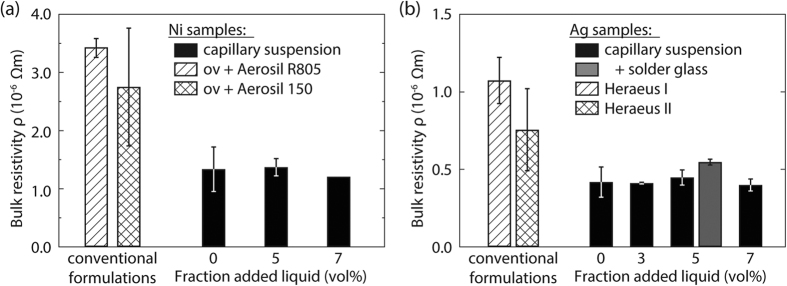
Bulk resistivity *ρ* for **(a**) nickel and **(b**) silver capillary suspensions (solids loading 29 vol%) compared to commercial paste formulations. For the nickel, the conventional formulations were prepared according to a Heraeus patent^21^ with an organic vehicle (ov) and two different thixotropic agents (Aerosil R805 and Aerosil 150) using the same particle fraction. Heraeus I and II are commerical silver pastes (solids volume fraction 90.0 and 90.5 wt%, respectively). In addition, a silver capillary suspension with 5 vol% water and 5 wt% solder glass is also shown (total solids loading 33 vol%). Variance in sheet thickness was measured and considered in the calculation of the bulk resistivity.

**Figure 5:**
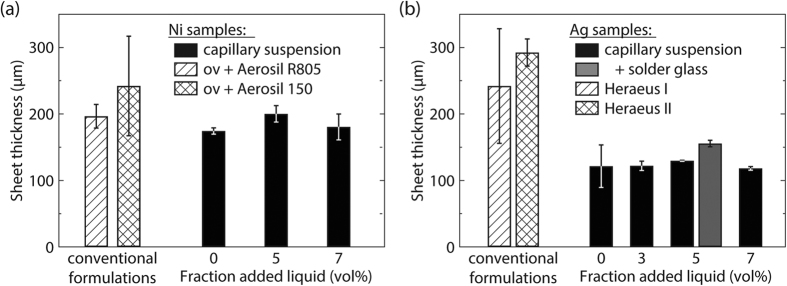
Final sheet thickness obtained for **(a**) nickel and **(b**) silver capillary suspensions, with 29 vol% particles in the original capillary suspension, compared to commercial paste formulations. For the nickel system, the conventional formulations were prepared according to a patent^21^ using an organic vehicle (ov) and two different thixotropic agents (Aerosil R805 and Aerosil 150) using the same particle fraction. Heraeus I and II are commercial silver pastes (solids volume fraction 90.0 and 90.5 wt%, respectively). In addition, data for a silver capillary suspension with 5 vol% water and 5 wt% solder glass is also shown (total solids loading 33 vol%).

**Table 1:** Particles and solvent combinations used for capillary suspension formulations described in this paper along with their interfacial tension.

Bulk phase	Secondary liquid	Interfacial tension *Γ*	Network type	
		[mN/m]		
Nickel				
Terpineol	Water	8.5	Capillary state	
Paraffin oil	Water	48.0^36^	Capillary state	
Paraffin oil	DMF/water (1:1)	15.6	Capillary state	
Paraffin oil	DMF	6.0	Capillary state	
Silver				
Terpineol	Water	8.5	Capillary state	

All of the capillary networks were in the capillary state where the secondary liquid does not preferentially wet the particles.

**Table 2:** Particles and solvents used for the capillary suspension formulations along with the product and supplier names.

Component name	Particle size according to supplier	Supplier
Solids		
Nickel spheres * Nickel powder 3–7 μm*	3–7 μm	Alfa Aesar (Karlsruhe, Germany)
Silver particles*Silverpulver GE C50*	5–15 μm	Doduco (Pforzheim, Germany)
Solder glass*lead-free solder glass 8470*	x_50 _≤ 12 μm, x_99 _≤ 63 μm	SCHOTT AG (Mainz, Germany)
Nonpolar solvents		
Terpineol* mixture of isomers, anhydrous*		Sigma Aldrich (Steinheim, Germany)
Paraffin oil*low viscosity paraffin oil*		Carl Roth (Karlsruhe, Germany)
Polar solvents		
N,N-Dimethylformamide (DMF)		VWR Chemicals (Darmstadt, Germany)
Distilled water		
